# Supplementary material for: Some New Aspects of Genetic Variability in Patients with Cutaneous T-Cell Lymphoma
Source: Genes (Basel). 2022 Dec 18;13(12):2401. doi: 10.3390/genes13122401 (PMC9778129; doi:10.3390/genes13122401)
Supplement: Supplementary file 1 [file genes-13-02401-s001.zip › genes-2057369-supplementary.pdf]

Table S1. Demographic characteristics of CTCL patients

Total/men	75/44
Age [years]	57,8±11.6
BMI [kg/m^2]	28.3±5.0
Smokers (%)	25 (33%)
Diabetes (%)	9 (12%)
Hypertension (%)	26 (35%)

Table S2 Staging of CTCL patients included in the study

Sex	Stage PP	Stage IA	Stage IB	Stage IIA	Stage IIB	Stage IIIA	Stage IIIB	Severity*
Men (N=44)	14	11	13	0	3	2	1	6/38 (16 %)
Women (N=31)	6	10	6	3	3	1	2	6/25 (24 %)
All	20	21	19	3	6	3	3	12/63 (19 %)

PP-parapsoriasis

*Proportion of (IIB+IIIA+ IIIB) to (PP+IA+IB+IIA)

Figure S1. Survival of the patients. Kaplan-Meier survival curve

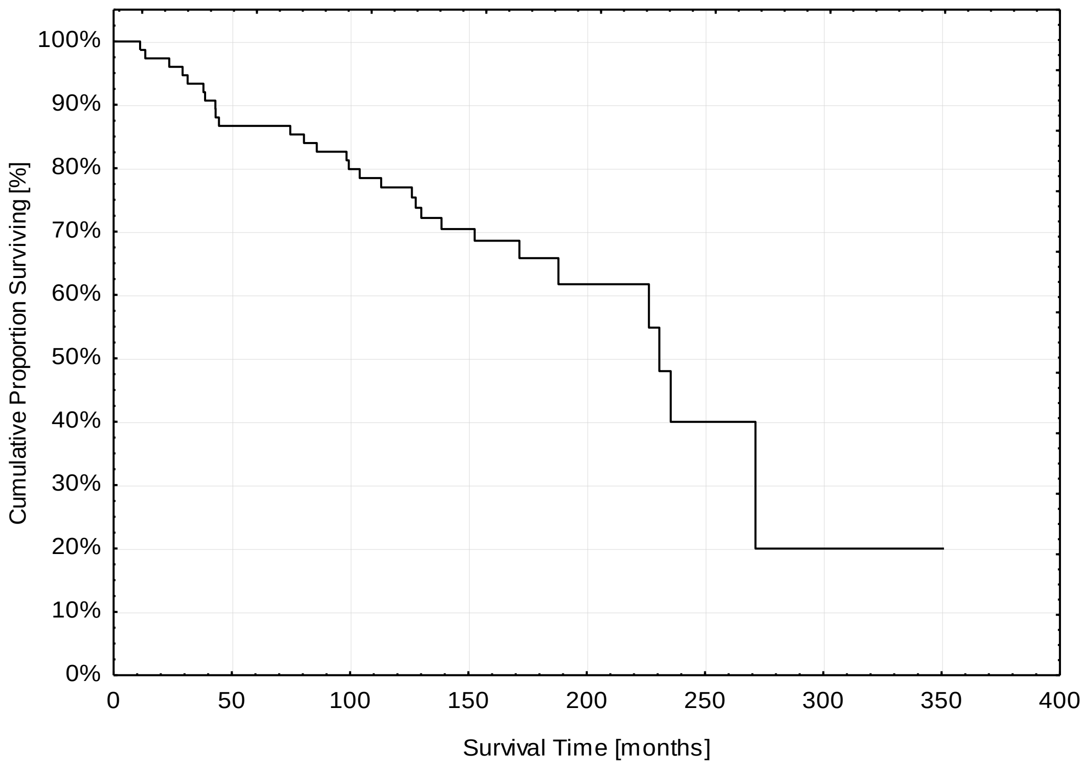

Table S3: Examined polymorphisms included to the study

Polymorphism	rs	MAF (%)*
Endothelin 8002 G/A	rs2071942	0.17
ACE_I/D	rs1799752	0.45
AGT M235T	rs699	0.45
AGT -6A/G	rs5051	0.43
Leptin -188 C/A	rs791620	0.05
Leptin -2548 A/G	rs7799039	0.44
Lepr MspI	rs1137101	0.49
Adiponectin 45 T/G	rs2241766	0.17
POMC RsaI	rs3754860	0.33
POMC 1032 C/G	rs1009388	0.28
MMP2 -1575 G/A	rs243866	0.25
MMP2 -1306 C/T**	rs243865	0.24
MMP2 -790 T/G	rs243864	0.26
MMP2 -735 C/T	rs2285053	0.12
TNFb_NcoI	rs909253	0.33
IL-6 -174 C/G	rs1800795	0.46
IL-6 Fok -597 A/G	rs1800797	0.43
MDR Ex21 2677***	rs2032582	0.45; 0.02
MDR Ex26 3435 C/T	rs1045642	0.41

*MAF (minor allele frequncy

** not in the Hardy-Weinberg equilibrium

*** Triallelic SNP; 4 genotypes: TT, GT, GG, GA; ratio G: T: A = 53 % : 45 % : 2%;

Table S4: The hazard ratios for three chosen polymorphisms and p-values in different inheritance models in patients with T-cell lymphoma

Model	Dominant		Codominant - Additive	
Polymorphism	HR (95% CI)	p-value	HR (95% CI)	p-value
MMP2 -790 (rs243864)	NS	NS	NS	NS
TNFb_NcoI (rs909253)	3.16 (1.14-8.71)	0.026*	1.96 (1.00-3.84)	0.049*
MDR Ex21 2677 (rs2032582)	0.35 (0.14-0.87)	0.024*	0.45 (0.24-0.86)	0.015

* Insignificant after Bonferroni correction

Figure S2. Survival of patients with different genotypes in MDR Ex21 2677 (rs2032582) - unadjusted

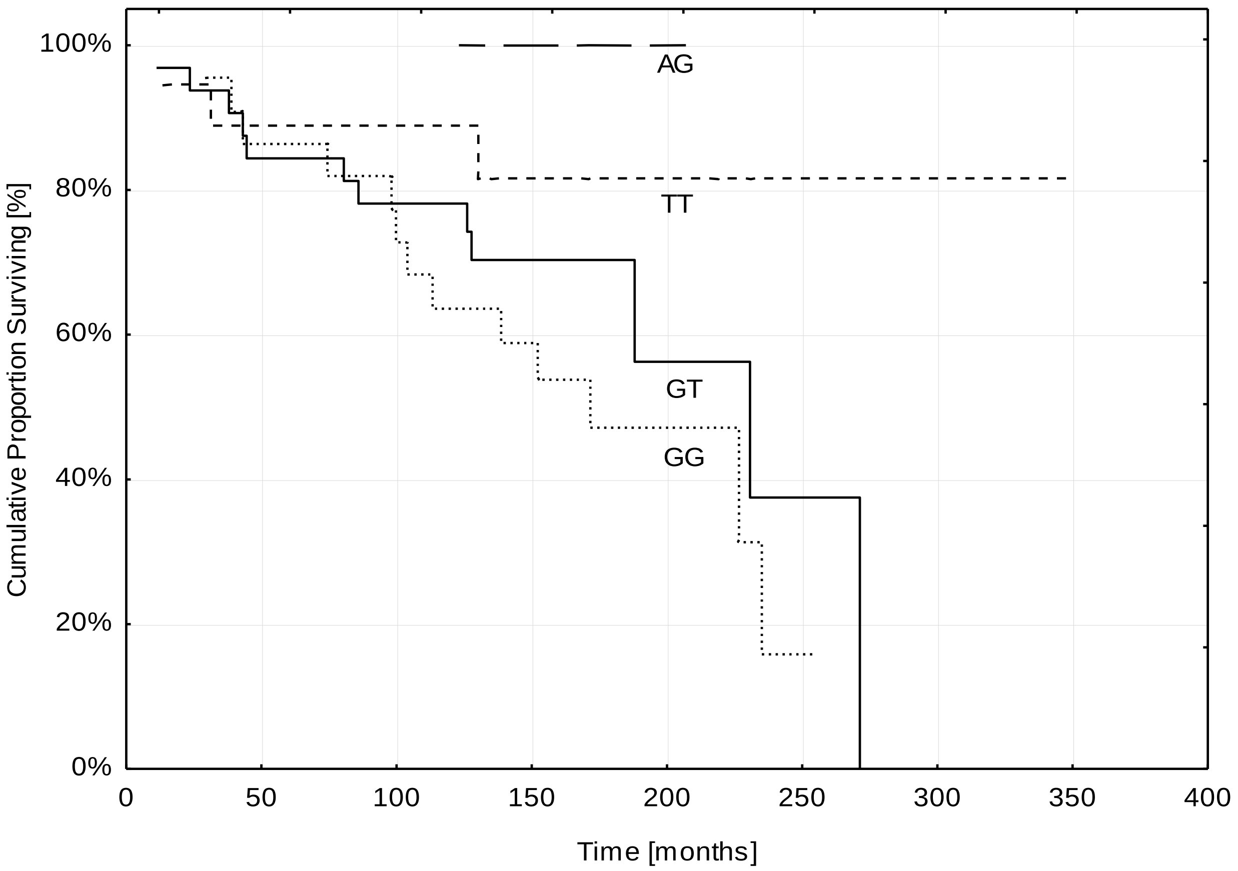

Table S5: MDR Ex21 2677 effect on the overall survival after adjustment for age, sex, staging, smoking status and treatment

Genotype	HR (95% CI)*	P-value
TT	0.020 (0.05-0.83)	0.027
GT	NS	NS
AG**	NS	NS

* After adjustment for age, sex, staging, smoking status and treatment

** Only 3 carriers of this genotype

Table S6: Multivariate stepwise Cox regression model including clinical and genetic aspects of T-cell lymphoma survival in Czech population

Factor	HR (95% CI)	P-value
Age at admission	4.35 (2.54–7.46) per 10 years	$9.\ 10^{-8}$
Sex (male vs. female)	3.20 (1.29–7.94)	0.012
Clinical stage (IIB+IIIA+IIIB) vs. (PP+IA+ IB+IIA)	3.25 (1.28–8.26)	0.013
MDR Ex21 2677 (rs2032582) genotype	0.51 (0.28–0.93)<br>per each T allele	0.027
